# Physical Activity, Body Composition, Physical Fitness, and Body Dissatisfaction in Physical Education of Extremadura Adolescents: An Exploratory Study

**DOI:** 10.3390/children11010083

**Published:** 2024-01-10

**Authors:** María Isabel Moreno-Díaz, Miguel Vaquero-Solís, Miguel Ángel Tapia-Serrano, Pedro Antonio Sánchez-Miguel

**Affiliations:** 1 Department of Didactics of Musical, Plastic and Corporal Expression, Faculty of Teacher Training, University of Extremadura, 10003 Cáceres, Spain; mmorenoag@alumnos.unex.es (M.I.M.-D.); mivaquero@alumnos.unex.es (M.V.-S.); pesanchezm@unex.es (P.A.S.-M.); 2 Faculty of Health and Sport Sciences, University of Zaragoza, 22002 Huesca, Spain

**Keywords:** physical education, cardiorespiratory fitness, body image, body mass index, adolescents

## Abstract

In recent years, physical activity levels among youths have declined significantly. This has led to a decline in adherence to physical activity recommendations. In this sense, physical education offers an ideal environment that contributes positively to improving adherence to physical activity recommendations, as it teaches students movement-related skills and knowledge. The objective of the present research was to investigate the relationship between physical activity levels, body composition, fitness in Physical Education, and body dissatisfaction levels, and to analyse sex differences in relation to the study variables. The sample was formed of 1166 participants from the 1st and 2nd secondary compulsory education, of which 642 were boys (age 13.16 ± 0.91), and 524 girls (age 13.08 ± 0.85). The measure of physical activity was the Physical Activity Questionnaire for Adolescents (PAQ-A). Body composition was assessed using weight and height to calculate their body mass index. Cardiorespiratory capacity was assessed using the Course-Navette test in Physical Education lessons. The results showed the relationship between physical activity and body mass index, cardiorespiratory capacity, and body satisfaction. It was also confirmed that higher levels of physical activity were associated with a lower body mass index, improvements in cardiorespiratory fitness, and lower levels of body dissatisfaction to a greater extent in boys than in girls. The study concludes that improvements in the study variables were associated with increased physical activity. In addition, it seems necessary to promote healthy lifestyles in physical education lessons, especially during adolescence, as they could serve as a gateway for the improvement of health-related fitness in future generations. Increasing the amount of physical activity among young people is vital. Therefore, it would be essential to develop intervention programs in physical education classes, especially during adolescence, aimed at promoting and increasing physical activity and its benefits.

## 1. Introduction

In human development, childhood and adolescence are two fundamental stages. The current and future state of health will be positively affected by developing, acquiring, and maintaining certain healthy behaviours during these stages [1,2]. During the early stages of life, especially in childhood and adolescence, a multitude of behavioral, cognitive, physical, and physiological changes occur [3]. Among these changes are those related to physical condition and body composition. These changes, as well as the acquisition, or not, of active healthy habits, are of great relevance in adulthood and even in old age [2].

In relation to healthy lifestyle habits, one of the most important which has decreased in recent years is physical activity [4]. This concept is defined as any physical movement performed by skeletal muscles that requires the expenditure of energy. Physical activity refers to all movement, including leisure, getting to and from places, and working. Both moderate and vigorous physical activity improve health [5]. According to the World Health Organization [6], obesity is a global epidemic with serious health consequences. Given this aspect, the World Health Organization recommends carrying out at least 60 min a day of physical activity of moderate to vigorous intensity for children and adolescents [6]. Accordingly, it is shown that a higher level of physical activity is relationship with a lower likelihood of being overweight or obese [7,8]. Moreover, greater physical, psychosocial, and cognitive benefits are associated with regular physical activity [9]. In this sense, physical education, which provides students with skills and knowledge related to physical activity, is an ideal environment for promoting physical activity, as well as training in physical activity skills that can be applied beyond the academic setting [10].

Otherwise, and following the healthy habits line, anthropometric aspects have been treated as an important factor to consider. In relation to body parameters, a previous study [11] has shown that having higher levels of body mass index (body mass index: defined as an indicator of body density as determined by the body weight to height ratio) is related to lower physical activity levels due to reduced energy expenditure. Furthermore, psychological problems such as low self-esteem, changes in body image, and difficulties with peer relationships are more common in obese children and adolescents [12]. Further, it is important to note that obese children and adolescents have been shown to be up to five times more likely to become obese in adulthood compared to those who are not obese when growing up [13].

Furthermore, and related to positive consequences in children and adolescents, regular physical activity has also been shown to improve fitness in children and adolescents [14]. This concept is defined as the ability to perform physical activity and/or exercise without excessive fatigue, and this term is identified as an integrated measure of all the functions involved in participation in physical activity or exercise [15]. Such is the importance of physical activity in the physical fitness of adolescents that it has been shown that a low level of cardiorespiratory fitness is a major cardiovascular risk factor, compared to other body composition parameters such as overweight and obesity [16]. Therefore, a high level of physical fitness means a good physiological response and, as a result, a lower risk of suffering from cardiovascular diseases [17]. Nevertheless, several factors of physical fitness, such as cardiorespiratory fitness and strength, have been declining over the last few decades [18]. As physical fitness tends to decline from early childhood to adulthood, this is a public health concern.

In accordance with all this, childhood, and especially adolescence, is a key period for the improvement and/or integration of body image [19]. Mainly this stage is key to avoiding body dissatisfaction. Body dissatisfaction is the negative evaluation an individual has of their own body, including judgements about size and shape, and a perceived discrepancy between their actual and ideal body type [20].

In relation to this, it is widely proven that children and adolescents are much more dissatisfied when their body mass index increases [19], fundamentally between the ages of 15 and 18, which is when it manifests more due to the passage of time and pubertal stage. This concept is widely marked at a cultural level, but there are aspects that can help its acceptance, as is the case of performing physical activity among school-age young people [21]. Avoiding high body dimensions in boys and girls is essential, since it avoids major maladaptive outcomes such as disordered eating and low self-esteem [22]. However, regarding psychosocial benefits, previous studies have shown that increased physical activity is positively associated with high body satisfaction [23,24]. Previous meta-analysis suggests that objective changes in physical fitness, perceived changes in physical fitness and, self-efficacy are three mechanisms that may promote the positive association between physical activity and body image [25]. Regarding the associations established jointly among physical activity, body mass index, and body satisfaction, people with higher levels of physical activity were found to have a better self-concept in the study by Trejo et al. [26]. Finally, and regarding sex, previous studies carried out in adolescents have shown that boys practice more physical activity than girls, showing differences in their body composition, physical fitness, and body satisfaction [21,22,27,28].

Previous studies have related different parameters to physical activity in isolation, whereas in the present research we relate all the parameters together in relation to the physical activity levels of adolescents. Moreover, we consider that it is a good exploratory study to continue investigating in the future. Therefore, based on the above, the present study establishes as its main objective (1) to know the association between physical activity with body composition (using body mass index), physical fitness (by cardiorespiratory fitness), and body dissatisfaction. The secondary objectives were (2) to identify the differences between the sexes for each of the above variables; and (3) test to what extent physical activity can predict physical fitness in adolescents. In relation to the objectives and based on previous studies, the following hypotheses were proposed:

**Hypothesis** **1 (H1).** *There will be significant relationships between the study variables (physical activity, body mass index, physical fitness, and body dissatisfaction).*

**Hypothesis** **2 (H2).** *Boys will obtain higher physical activity scores and physical fitness, and lower body composition scores and body dissatisfaction than girls.*

**Hypothesis** **3 (H3).** *Adolescents with higher levels of physical activity would show a higher level of physical fitness.*

## 2. Material and Methods

### 2.1. Participants and Design

This cross-sectional study was developed as part of the Physical Activity and Promotion of Health-Related Behaviours in Adolescents project [29]. Baseline data were collected from adolescents in Extremadura (Spain) between March 2018 and June 2019. In total, 1166 participants from 22 secondary schools were recruited in this study, of which 642 were boys (*M* = 13.16; *SD* = 0.91) and 524 were girls (*M* = 13.08; *SD* = 0.85). The type of sampling was convenience-based depending on the availability of the schools to collaborate in the research. Participants were selected based on their age. Participation within the school was voluntary and only participants who did not want to participate were excluded. If any participant had any of the study items missing, they were also excluded. First, the questionnaire on levels of physical activity and body dissatisfaction was administered. Once conducted, we valued the anthropometric measures of the participants and then the physical tests were carried out. At that time, during school hours, we collected the data so we had the results of the questionnaires in less than an hour while the physical and anthropometric tests took longer, approximately two to three hours. When we had collected all the information from the same school, we visited another. The investigation was approved for data collection by the Ethics Committee of the Faculty of Teaching and Teacher Education of the University of Extremadura. This research was carried out in accordance with the ethical guidelines of the Declaration of Helsinki (89/2016).

### 2.2. Instruments

*Anthropometric measures*. Adiposity parameters were assessed using standardised anthropometric techniques. Each measurement was performed twice, and the average was recorded. Weight was measured with an electronic scale (SECA 701; range, 0.05–220 kg; precision, 0.05 kg) and height was measured using the Frankfort Plane and a telescopic measuring rod (SECA 220; range, 85–200 cm; precision, 1 mm). After measuring weight and height, the body mass index (BMI) was calculated by dividing the weight in kilograms by the square of the height in meters.

*Physical activity.* The physical activity was estimated using the Physical Activity Questionnaire for Adolescents (PAQ-A) previously adapted and validated into Spanish by Martínez-Gómez et al. [30]. The validity and reliability of this scale for assessing physical activity levels in Spanish adolescents is supported by its correlation with total physical activity (r = 0.39), and moderate and vigorous physical activity (r = 0.34) as assessed by accelerometer, as well as its alpha coefficient (α = 0.79) and intraclass correlation coefficient (ICC = 0.71) [30,31]. The Cronbach’s alpha for this scale was also found to be 0.79 in the present investigation. The scale comprises of nine items that evaluate physical activity participation over the past seven days at various times, such as during physical education classes, school breaks, lunchtime, after school, evenings, and weekends. Each response is scored on a 5-point scale ranging from 1 to 5. The physical activity score is calculated by averaging all responses. Higher scores indicate higher levels of physical activity.

*Cardiorespiratory fitness*. The Course-Navette test was used to assess cardiorespiratory fitness [14]. This is a field test that involves running from one line to another which is 20 metres away, and gradually increasing the speed based on a sound signal. The initial speed is 8.5 km/h, and it increases by 0.5 km/h. The test ends when the participant fails to reach the finish line at the same time as the audio signal for the second consecutive time, or when the competitor stops due to faulty equipment. A loudspeaker to reproduce the sound signal and a 20-metre non-slip surface are required. The test has been shown to be valid and reliable in children and adolescents. To calculate cardiorespiratory fitness, the Leger [32] formula was used, and its measurement is in VO_2max_ [32].

*Body dissatisfaction*. Body dissatisfaction was assessed with the body image dimensional assessment instrument [33]. This instrument has been previously validated in Spanish adolescents [33]. The body image dissatisfaction assessment (BIDA) is a questionnaire comprising four items. Students answer questions about four silhouettes related to their sex and age, allowing them to quantify their level of body dissatisfaction (BD), sexual body dissatisfaction (SxBD), and comparative body dissatisfaction (CBD). The text adheres to conventional structure and formatting features, including consistent citation and footnote style. No changes in content have been made. The final body dissatisfaction index (BDI) is calculated based on the difference between the actual and ideal body image. The language used is clear, concise, and objective, with a formal register and precise word choice. The logical structure and causal connections between statements are maintained, and the text is free from grammatical errors, spelling mistakes, and punctuation errors. SxBD indicates the difference between the subject’s current body image and their ideal figure for the opposite gender. CBD expresses the difference between the subject’s current body image and that of many same-sex, same-age people. The scores for the three items were expressed as percentages, ranging from −100% to 100%. Positive scores indicate greater body dissatisfaction, while lower scores indicate less body dissatisfaction. The BDI score was calculated as the mean of the absolute values of SxBD, ScBD, and CBD.

*Covariates*. Participants self-reported their age (in years), sex (male/female), and socioeconomic status. Children’s and adolescent’s socioeconomic status was measured using the Family Affluence Scale-III (FAS III; [34]). A socioeconomic status score ranging from 0 to 9 was calculated based on the responses to four questions.

### 2.3. Procedure

Letters were sent to parents and head teachers explaining the nature and purpose of the study. Both parents and participants were given written informed consent, which explained the aims and nature of the study. This consent form was signed by the parents and/or legal guardians and by the participants themselves for participation in the research. All students who did not provide informed consent were not included. To carry out the evaluations, the research team travelled to the school. The evaluations were conducted by experts in physical activity and sports. The measurements were created by the same research team, with each member specialising in a specific test to minimise the possibility of measurement errors. The assessments were carried out on the same day at school, always starting with the anthropometric variables, later the questionnaires were provided to analyse the perceived or self-reported variables, and finally the physical variables were carried out. All this with the purpose of homogenising the tests, avoiding any contamination in the individuals or alterations that could affect the normal collection of data.

### 2.4. Analysis of Data

Descriptive statistics (i.e., mean scores and standard deviations), and Pearson correlations were calculated for continuous variables. To test whether the data were normally distributed, the Kolmogorov-Smirnov test was also performed. SPSS version 23.0 for Windows (IBM) was used to perform these analyses. The Pearson coefficient was also used to calculate correlations between study variables.

Finally, the statistical package Mplus 7.0 [35] was used to assess the predictive capacity SEM structural equation model (SEM) of fitness, body mass index, and body image dissatisfaction on cardiorespiratory fitness. Likewise, to ensure a proper model fit, we used the following goodness-of-fit indices as reference points: Minimum Residual Likelihood (MRL), Comparative Fit Index (CFI), Tucker–Lewis Index (TLI), Standardised Root Mean Square Residual (SRMR), and Root Mean Square Error of Approximation (RMSA). Regarding the CFI and TLI, values above 0.90 are considered suitable indicators of good fit [36]. However, for SRMR and RMSA coefficients, values below 0.60 are considered correct [36]. Although other studies have demonstrated acceptable values between 0.60 and 0.80 [37].

## 3. Results

Table 1 presents the descriptive statistics of the variables studied. Independent samples *t*-tests revealed significant differences in physical activity between males and females (*p* > 0.05) and in cardiorespiratory fitness (*p* > 0.05), but not for body mass index and body dissatisfaction, where no differences were found. They presented significant differences, although average scores were slightly higher than the male sex in body dissatisfaction.

Bivariate correlations for the study variables are shown in the same table. In this sense, significant positive correlations were shown between body fitness and cardiorespiratory fitness (r = 0.33; *p* < 0.05), and significant negative correlations with body dissatisfaction (r = −0.10; *p* < 0.05). On the other hand, body mass index was significantly positively associated with body dissatisfaction (r = 0.58; *p* < 0.05), and significantly negatively associated with cardiorespiratory fitness (r = −0.28; *p* < 0.05).

**Figure 1:**
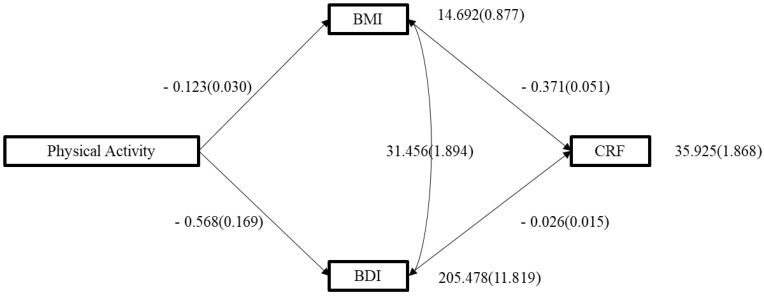
Structural equation model predictor of cardiorespiratory fitness. *Note.* BMI: Body mass index; BDI: Body dissatisfaction index; CRF: Cardiorespiratory fitness.

Finally, to assess the predictive ability of physical activity on cardiorespiratory capacity through body dissatisfaction and body mass index, the following predictive model (Figure 1) was posited, but it did not exhibit good fit indices: MRL_2 = 112.541, *p* < 0.05, CFI = 0.63, TLI = −0.11, SRMR = 0.15, and RMSA = 0.22. Therefore, a decision was made to restructure the model based on modification indices. These suggested modifications led us to adjust the model concerning the relationships between body mass index and body dissatisfaction. These modifications resulted in the model (Figure 1) displaying good fit indices: MRL_2 = 302.755, *p* < 0.05, CFI = 0.99, TLI = 0.98, SRMR = 0.02, and RMSA = 0.02, suggesting that both body dissatisfaction and BMI negatively predict cardiorespiratory capacity.

## 4. Discussion

The objective of this study was to understand the association between physical activity and body composition, physical fitness, and body dissatisfaction. The first hypothesis indicated that there will be significant correlations between the study variables (physical activity, body mass index, physical fitness, and body dissatisfaction). The results confirmed the hypothesis, except for body dissatisfaction and body mass index which were negatively related, but not significantly. In line with the above, Ruiz et al. [14] showed significant associations between levels of body dissatisfaction and physical fitness in children and adolescents. In the same way and as in the present study, Prieto’s [38] research negatively associates physical activity and body mass index, but in this case and unlike our study, the association was significant. Moreover, these results are in line with those obtained by Coelho et al. [39], who concluded that four days per week of physical activity can reduce or regulate body dissatisfaction.

Additionally, Bassett-Gunter et al. [25] conducted a meta-analysis that provided scientific evidence supporting the critical role of three mechanisms in explaining the association between physical activity and body image: goal-directed changes in physical activity, perceived changes in physical fitness, and changes in self-efficacy. Considering this research and others, such as Santana et al. [40], higher levels of physical activity led to a decrease in body mass index, resulting in better physical condition and lower levels of body dissatisfaction. Furthermore, physical activity in early ages is important as it is associated with higher levels of cardiopulmonary fitness, according to recent systematic reviews [39]. In line with these results, a three-year longitudinal study found that an increase of 6.9 min per day of physical activity was associated with an increase in the mean distance of the 20-metre walk test from 968 to 1070 m [40]. Therefore, accumulating minutes of physical activity per day can be shown to improve cardiorespiratory fitness [41,42,43].

Concerning the second hypothesis, it was suggested that boys would achieve better scores in physical activity, body composition, physical fitness, and body satisfaction than girls. The results partially confirmed the hypothesis, as higher levels of physical activity and cardiorespiratory fitness were achieved. However, no significant differences were observed for body satisfaction and body mass index. In accordance with this, the study by Muros et al. [44] is consistent with these results. This research stated that no differences in body mass index were found regarding sex. Likewise, body mass index scores were lower in girls, as was shown in the research by Ramos, Rivera, and Moreno [45], which showed that boys have higher body mass index than girls. Therefore, the second hypothesis could be confirmed in all its parameters except body mass index. The higher the value of an individual’s cardiorespiratory capacity, the greater the amount and intensity of aerobic work they can perform, surely a consequence of having higher levels of physical activity. That is, a better motor capacity may be a consequence of them being faster individuals. In line with the results of the present study, it has been shown that boys are faster than girls. Likewise, studies such as that of Mayorga et al. [46] had results similar to the present study.

On the other hand, in relation to the sex differences in physical activity levels, the study shows that the participants who conducted more physical activity were boys, showing significant differences between both sexes. This result is consistent with that found by Zurita-Ortega et al. [47], in which significant relationships between physical activity levels in terms of sex were found in their results. Along these lines, the results of Saucedo et al. [48] are consistent with this study and show that boys were more physically active than girls. 

This research also revealed differences, although not significant, have been obtained between body satisfaction in boys and girls, presenting higher scores in boys. According to results of the present research, the previous studies by Cachón-Zagalaz et al. [49] and Palomares-González et al. [50] obtained results similar and concluded that boys had higher body satisfaction scores than girls. In addition to this, the study conducted by Sánchez-Miguel et al. [21] found that boys who engage in higher levels of physical activity are more likely to be satisfied with their physical appearance. To conclude the second hypothesis, significant differences in cardiorespiratory fitness were found between boys and girls. Thus, the higher the cardiorespiratory fitness value of a student, the greater the amount and intensity of aerobic work they can perform, surely a consequence of having felt more body dissatisfied. That is, a better score in cardiorespiratory fitness may be a consequence of being faster individuals. In line with the results of the present study, it has been shown that boys have better oxygen consumption than girls. Similarly, the study by Gómez-Campos et al. [51] show similar results to the present work since they demonstrated that adolescents who practiced physical activity regularly had better levels of cardiorespiratory fitness.

Regarding our hypothesis, our study has shown that there is a positive and significant relationship between physical activity and physical fitness. Additionally, our findings also revealed negative relationships between physical activity, body dissatisfaction, and BMI. This finding aligns with the findings in the studies by Neil-Sztramko [52] and Fairclough [53], which show that interventions on physical activity conducted in the school environment can impact both physical fitness and body dissatisfaction, as well as body mass index. This could be explained through the effects of multicomponent interventions aimed at increasing the amount of daily physical activity in children and adolescents, where strategies such as active breaks or active recess periods might affect physical fitness or BMI.

Despite the important findings, there are some limitations to the present research. Firstly, as a cross-sectional investigation, it is not possible to establish cause-effect relationships of the results, so the cause-effect relationship of physical activity on body mass index, cardiorespiratory fitness and body dissatisfaction cannot be demonstrated. Secondly, the level of physical activity has been self-reported by the students, which does not allow us to know exactly the amount physical activity of the children and adolescents and can lead to biases in the evaluation of physical activity. Thirdly, the non-randomisation of the sample may introduce certain biases in its interpretation. To address these limitations, the use of objective tools such as accelerometers to measure physical activity more accurately would be useful in future research. Furthermore, these instruments could be used to test for differences between moderate and vigorous activity in relation to adolescents’ body mass index, cardiorespiratory fitness, and body dissatisfaction. Furthermore, in the future, it would be interesting to explore the relationship between the analysed variables from a qualitative perspective. This would involve assessing the acceptance of one’s body based on individual criteria, as well as the potential for physical activity and fitness improvement. Such an approach would provide a more comprehensive assessment of these variables and aid in the development of programs aimed at improving health outcomes among children and adolescents.

## 5. Conclusions

In conclusion, the results showed that boys who exercised had lower body mass index and lower body dissatisfaction compared to girls who exercised. This study provides evidence of the impact of physical activity on body composition, physical fitness, and body dissatisfaction in children and adolescents. It is important to note that higher levels of physical activity are associated with better body composition and physical fitness, as well as lower levels of body dissatisfaction. It is important to recognise that there may be individual variations in the effects of physical activity on different adolescents. Increasing physical activity among adolescents is vital due to the associated physical and psychological benefits. Physical education in school provides an ideal environment to promote physical activity and improve young people’s perceptions and body image. Therefore, it would be logical to develop intervention programs, particularly during adolescence, to promote and increase physical activity. This is due to the benefits associated with this behaviour.

## Figures and Tables

**Table 1:** Descriptive statistics of the study variables.

Study Variables	Total	Boys	Girls	*F*	*p*	Bivariate Correlations			
	*M ± SD*	*M ± SD*	*M ± SD*			1	2	3	4
1. Physical activity	2.21 ± 0.50	2.31 ± 0.34	2.10 ± 0.40	2.51	<0.001	-	−0.06	−0.10 *	0.33 **
2. BMI	21.3 ± 3.83	21.3 ± 2.85	21.2 ± 3.02	3.39	0.71	-	-	0.58 **	−0.29 **
3. Body dissatisfaction	3.17 ± 14.3	3.05 ± 12.28	3.30 ± 13.38	1.64	0.77	-	-	-	−0.22 **
4. Cardiorespiratory fitness	42.7 ± 5.93	44.7 ± 5.42	40.3 ± 6.02	71.9	<0.001	-	-	-	-

*Note.* * *p* < 0.05; ** *p* < 0.05; *M*: Media; *SD*: Standard deviation; BMI: Body Mass Index.

## Data Availability

The data presented in this study are available on request from the corresponding author. The data are not publicly available due to privacy and ethical restrictions.
